# Discovery of a diaminoquinoxaline benzenesulfonamide antagonist of HIV-1 Nef function using a yeast-based phenotypic screen

**DOI:** 10.1186/1742-4690-10-135

**Published:** 2013-11-14

**Authors:** Ronald P Trible, Purushottam Narute, Lori A Emert-Sedlak, John Jeff Alvarado, Katelyn Atkins, Laurel Thomas, Toshiaki Kodama, Naveena Yanamala, Vasiliy Korotchenko, Billy W Day, Gary Thomas, Thomas E Smithgall

**Affiliations:** 1 Department of Microbiology and Molecular Genetics, University of Pittsburgh School of Medicine, Bridgeside Point II, Suite 523, 15219, Pittsburgh, PA USA; 2 School of Medicine, Oregon Health and Science University, 97239, Portland, OR, USA; 3 Department of Structural Biology, University of Pittsburgh School of Medicine, 15261, Pittsburgh, PA USA; 4 Department of Pharmaceutical Sciences, University of Pittsburgh School of Pharmacy, 15261, Pittsburgh, PA USA

**Keywords:** HIV-1, Nef, Src-family kinases, Hck, Zap-70, MHC-I downregulation, Small molecule Nef antagonists

## Abstract

**Background:**

HIV-1 Nef is a viral accessory protein critical for AIDS progression. Nef lacks intrinsic catalytic activity and binds multiple host cell signaling proteins, including Hck and other Src-family tyrosine kinases. Nef binding induces constitutive Hck activation that may contribute to HIV pathogenesis by promoting viral infectivity, replication and downregulation of cell-surface MHC-I molecules. In this study, we developed a yeast-based phenotypic screen to identify small molecules that inhibit the Nef-Hck complex.

**Results:**

Nef-Hck interaction was faithfully reconstituted in yeast cells, resulting in kinase activation and growth arrest. Yeast cells expressing the Nef-Hck complex were used to screen a library of small heterocyclic compounds for their ability to rescue growth inhibition. The screen identified a dihydrobenzo-1,4-dioxin-substituted analog of 2-quinoxalinyl-3-aminobenzene-sulfonamide (DQBS) as a potent inhibitor of Nef-dependent HIV-1 replication and MHC-I downregulation in T-cells. Docking studies predicted direct binding of DQBS to Nef which was confirmed in differential scanning fluorimetry assays with recombinant purified Nef protein. DQBS also potently inhibited the replication of HIV-1 NL4-3 chimeras expressing Nef alleles representative of all M-group HIV-1 clades.

**Conclusions:**

Our findings demonstrate the utility of a yeast-based growth reversion assay for the identification of small molecule Nef antagonists. Inhibitors of Nef function discovered with this assay, such as DQBS, may complement the activity of current antiretroviral therapies by enabling immune recognition of HIV-infected cells through the rescue of cell surface MHC-I.

## Background

HIV-1 *nef* encodes a small myristoylated protein required for optimal viral replication and AIDS pathogenesis [1,2]. Deletion of *nef* from the HIV-related simian immunodeficiency virus prevents AIDS-like disease progression in rhesus macaques [3]. In addition, expression of the *nef* gene alone is sufficient to induce an AIDS-like syndrome in transgenic mice very similar to that observed upon expression of the complete HIV-1 provirus [4,5]. In humans, *nef* sequence variability and function correlate with HIV disease progression over the course of infection [6,7]. Indeed, long-term non-progressive HIV infection has been associated with *nef*-defective strains of HIV in some cases [8-10]. These and other studies identify the HIV-1 Nef accessory protein as a key molecular determinant of AIDS.

Nef lacks any known intrinsic enzymatic or biochemical function and instead exploits multiple host cell signaling pathways to optimize conditions for viral replication and AIDS progression [11,12]. Growing evidence identifies the Src family kinases (SFKs) as key molecular targets for Nef [13]. One important example is Hck, a Src family member expressed in macrophages that binds strongly to Nef via an SH3-mediated interaction [14,15]. Nef binding leads to constitutive Hck activation [16,17], which may be important for macrophage survival [18] and productive infection by M-tropic HIV [19]. In addition, activation of Hck, Lyn or c-Src is a critical first step in the downregulation of cell-surface MHC-I by Nef, which enables immune escape of HIV-infected cells [20-22]. Transgenic mice expressing a Nef mutant lacking a highly conserved PxxPxR motif essential for activation of Hck and other SFKs showed no evidence of AIDS-like disease [23]. When the Nef-transgenic mice were crossed into a *hck*-null background, appearance of the AIDS-like phenotype was delayed with reduced mortality [23]. These observations support an essential role for Nef interactions with Hck and other SFKs in multiple aspects of AIDS pathogenesis.

In this report, we describe the development of a yeast-based screen to identify inhibitors of Nef signaling through SFKs. First, we established that co-expression with Nef leads to constitutive activation of Hck in yeast by the same biochemical mechanism observed in mammalian cells. The active Nef:Hck complex induced growth arrest in yeast that was reversed with a known SFK inhibitor, providing a basis for a simple yet powerful screen for novel compounds. Using this system, we screened a small chemical library of drug-like heterocycles and identified a diaminoquinoxaline benzenesulfonamide analog that potently blocks Nef-dependent HIV replication and MHC-I downregulation. Docking studies and differential scanning fluorimetry assays support direct interaction of this compound with Nef as its mechanism of action. Small molecules that interfere with Nef-mediated downregulation of MHC-I molecules may represent powerful adjuvants to existing antiretroviral drugs by thwarting the viral strategy of immune evasion.

## Results

### Hck-YEEI models Csk-downregulated Hck in yeast

Previous work has shown that ectopic expression of active c-Src induces growth arrest in yeast [24-27]. Co-expression of C-terminal Src kinase (Csk), a negative regulator of SFKs [28], rescues Src-mediated growth suppression by phosphorylating the c-Src negative regulatory tail and repressing kinase activity [26,29-31]. Using a similar yeast-based system, we have previously shown that other members of the Src kinase family also induce yeast growth arrest in a Csk-reversible manner [29]. Co-expression of HIV-1 Nef selectively overcomes Csk-mediated negative regulation for Hck, Lyn, and c-Src, resulting in kinase re-activation and growth arrest [29]. These observations suggest that the yeast system may provide the basis for an inhibitor screen, as compounds which block Nef-induced SFK signaling are predicted to rescue cell growth.

**Figure 1:**
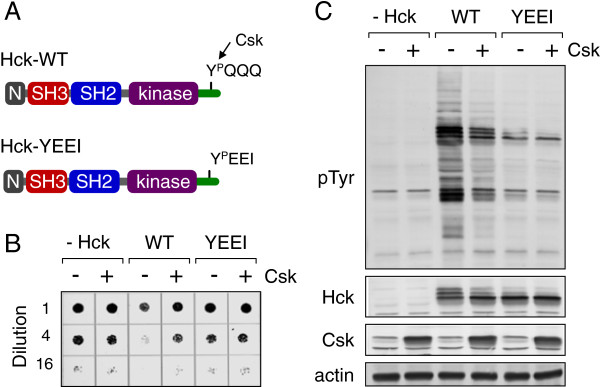
**Hck-YEEI models Csk-downregulated Hck in yeast. A)** Domain organization of wild-type (WT) Hck and Hck-YEEI. Both kinases consist of an N-terminal unique domain (N), SH3 and SH2 domains, a kinase domain and a negative regulatory tail with a conserved tyrosine phosphorylation site. In wild-type Hck, the tail sequence is Tyr-Gln-Gln-Gln (YQQQ), and requires Csk for phosphorylation. In Hck-YEEI, this sequence was modified to Tyr-Glu-Glu-Ile (YEEI), which allows for Csk-independent downregulation in yeast. **B)** Yeast cultures were transformed with expression plasmids for wild-type Hck (WT), Hck-YEEI (YEEI) or the empty expression plasmid (−Hck). Cells were co-transformed with expression vectors for Csk (+) or the corresponding empty vector (−) as indicated. Cells were spotted onto agar selection plates containing galactose as the sole carbon source to induce kinase expression and incubated for 3 days at 30°C. Cultures were spotted in four-fold dilutions to enhance visualization of the growth suppressive phenotype. Plates were scanned and yeast patches appear as dark circles. **C)** Immunoblots from cultures shown in part **B**. Transformed cells were grown in liquid culture in the presence of galactose at 30°C for 18 h. Protein extracts were separated via SDS-PAGE, and immunoblotted for tyrosine-phosphorylated proteins (pTyr) as well as for Hck, Csk, and actin as a loading control.

To simplify the yeast assay for chemical library screening, we substituted the sequence of the Hck negative regulatory tail with the high-affinity SH2-binding motif, Tyr-Glu-Glu-Ile (YEEI; Figure 1A). Previous work has shown that the YEEI modification results in autophosphorylation of the tail, leading to intramolecular engagement of the SH2 domain and downregulation of kinase activity in the absence of Csk [32]. Importantly, the X-ray crystal structure of this modified form of Hck (referred to hereafter as Hck-YEEI) is nearly identical to that of native Hck that has been down-regulated by Csk [32,33]. To determine whether the YEEI substitution was sufficient to downregulate Hck in yeast, wild-type Hck and Hck-YEEI were expressed in the presence or absence of Csk. Hck-YEEI failed to suppress yeast growth, and showed reduced kinase activity compared with wild-type Hck on anti-phosphotyrosine immunoblots of yeast cell lysates (Figure 1B,C). Co-expression of Csk reduced wild-type Hck kinase activity and reversed growth suppression, but had no effect on Hck-YEEI, as it is already auto-down-regulated. These results show that Hck-YEEI effectively models the behavior of Csk-downregulated wild-type Hck in yeast, supporting the substitution of Hck-YEEI for wild-type Hck plus Csk to model downregulated Hck in yeast.

### Nef activates Hck-YEEI in yeast by the same molecular mechanism observed in mammalian cells

**Figure 2:**
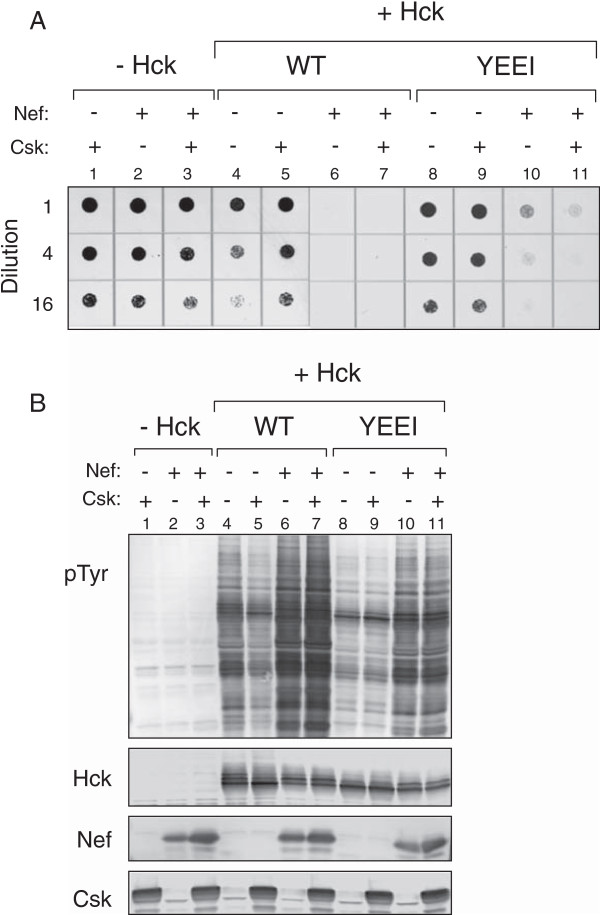
**Nef activates Hck-YEEI in the same manner as Csk-downregulated wild-type Hck in yeast.** Yeast cultures were co-transformed with expression plasmids for wild-type Hck, Hck-YEEI, Csk, and Nef in the combinations shown. **A)** Cultures were grown on galactose-agar plates and scanned as described in the legend to Figure 1. **B)** Immunoblots from cultures shown in panel **A**. Transformed cells were grown in liquid culture in the presence of galactose at 30°C for 18 h. Protein extracts were separated via SDS-PAGE, and immunoblotted for tyrosine-phosphorylated proteins (pTyr) and for Hck, Csk, and Nef. Note that the numbers in panel **A** correspond with the lanes in panel **B***.*

HIV-1 Nef activates Csk-downregulated Hck in yeast, leading to growth suppression [29]. To determine whether Nef activates Hck-YEEI in the same manner, yeast cultures were transformed with plasmids encoding wild-type Hck or Hck-YEEI in the presence or absence of Csk and Nef. Csk and Nef expression had no effect on yeast growth in the absence of Hck (Figure 2A, columns 1–3). Wild-type Hck suppressed yeast growth, and this effect was reversed upon co-expression of Csk as expected (columns 4 and 5). Nef strongly enhanced Hck-mediated growth suppression independently of Csk (columns 6 and 7) as observed previously [29]. Importantly, co-expression of Nef with Hck-YEEI also induced a strong growth suppressive effect which was unaffected by Csk (columns 8–11). Co-expression of Nef with wild-type Hck resulted in much stronger tyrosine phosphorylation of yeast proteins than observed with Hck alone or in the presence of Csk (Figure 2B, lanes 4–7). Nef produced a similar increase in the kinase activity of Hck-YEEI (lanes 8 and 10). The effects of Nef on yeast protein-tyrosine phosphorylation by wild-type Hck and Hck-YEEI were unaffected by Csk (lanes 7 and 11). In all cases, a strong inverse correlation was observed between Hck kinase activity and yeast cell growth. These data establish that Nef strongly activates Hck-YEEI and induces a growth-suppressive phenotype very similar to that observed with wild-type Hck. Note that all transformed yeast cultures grew in an identical fashion when grown on glucose medium, demonstrating that the growth suppressive effects are due to induction of the Nef and Hck proteins and not a general cytotoxic effect.

**Figure 3:**
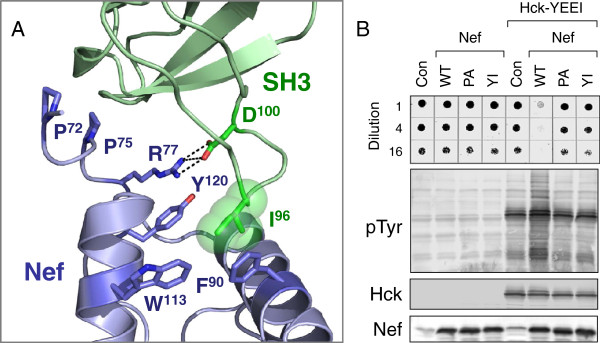
**Activation of Hck-YEEI in yeast requires the Nef PxxPxR motif and hydrophobic pocket. A)** Conserved features of the Nef:SH3 interface. The SH3 domain is shown in green, while Nef is colored blue. Side chains of conserved prolines in the Nef N-terminal region that contact the SH3 hydrophobic surface are shown (Pro72; Pro75) along with Arg77 which forms a salt bridge with SH3 Asp100. The SH3 domain RT loop Ile reside (Ile96; green spheres) interacts with several conserved residues that extend from the intersection of the Nef αA and αB helices to form a hydrophobic pocket (Phe90, Trp113, Tyr120). This model was produced using the crystallographic coordinates of Lee, et al. (PDB: 1EFN) [35]. **B)** Upper panel, left four lanes: growth of yeast cultures expressing wild-type Nef (WT), a Nef-PA mutant in which the PxxP motif is replaced by AxxA (PA), the Nef hydrophobic pocket mutant Y120I (YI), or no Nef (Con). The cultures shown in the right four lanes also co-expressed Hck-YEEI. All cultures were spotted and scanned as per the legend to Figure 1. Lower panels: Lysates from the yeast cultures shown in the top panel were immunoblotted with anti-phosphotyrosine antibodies (pTyr) as well as Hck, and Nef antibodies.

We next investigated whether the key structural determinants of Nef-induced Hck activation were functional in the yeast system. Nef binds to the Hck SH3 domain, disrupting its negative regulatory influence on the kinase domain [34]. To determine if Nef activates Hck-YEEI via this SH3 domain displacement mechanism in yeast, we substituted the prolines in the Nef P^72^xxP^75^xR motif essential for SH3 recognition with alanines (Figure 3A), and co-expressed this mutant (Nef-PA) with Hck-YEEI. In contrast to wild-type Nef, the Nef-PA mutant failed to activate Hck-YEEI and induce growth suppression (Figure 3B).

A second structural determinant of Nef interaction with SH3 involves a hydrophobic pocket formed by several conserved non-polar side chains in the Nef core (Phe90, Trp113, Tyr120; Figure 3A). These residues interact with SH3 Ile96, a residue unique to the RT loops of the Hck and Lyn SH3 domains [35] (Figure 3A). Substitution of Tyr120 within this Nef hydrophobic pocket with isoleucine (Nef-Y120I) disrupts Nef-mediated Hck activation in a rodent fibroblast model system [36]. Similarly, Nef-Y120I was unable to activate Hck-YEEI in yeast and failed to produce growth suppression (Figure 3B). These data show that Nef recognizes and activates Hck-YEEI in yeast through the same mechanism observed in mammalian cells.

### Chemical inhibition of Nef:Hck-YEEI activity restores yeast growth

**Figure 4:**
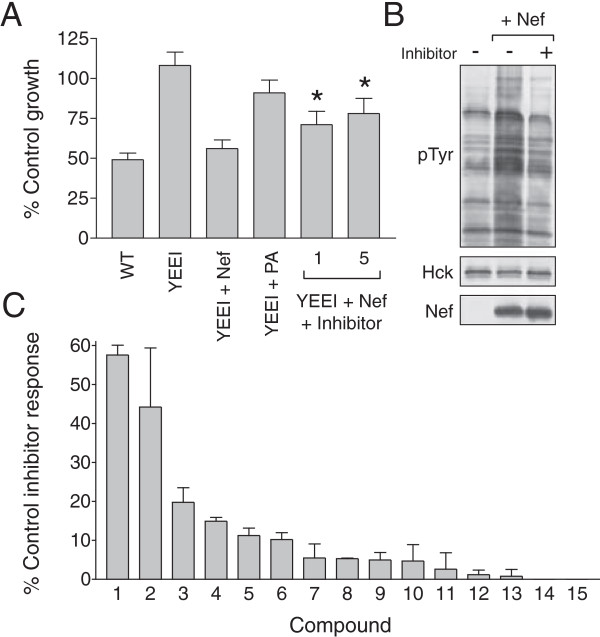
**Identification of inhibitors of Nef:Hck-YEEI signaling in yeast. A)** Assay validation. Liquid cultures of yeast expressing wild-type Hck (WT), Hck-YEEI (YEEI), and Hck-YEEI plus wild-type Nef or the PA mutant were grown in 96-well plates for 22 h at 30°C. Cultures expressing Hck-YEEI and Nef were also grown in the presence of the broad-spectrum SFK inhibitor A-419259 at 1 and 5 μM under the same conditions. Growth was recorded as change in optical density at 600 nm, and data are normalized to the percentage of growth observed relative to cells transformed with the empty expression plasmids. Each condition was repeated in triplicate, and the bargraph shows the mean percentage of control growth ± S.D. The statistical significance of the values obtained with Hck-YEEI plus Nef alone was compared to the same cultures grown in the presence of 1 or 5 μM A-419259 (Student’s t-test; *p = .01). **B)** Yeast cultures expressing Hck-YEEI alone or Hck-YEEI plus Nef in the presence (+) or absence (−) of 5 μM A-419259 were grown in liquid medium in the presence of galactose at 30°C for 18 h. Protein extracts were separated via SDS-PAGE, and immunoblotted for tyrosine-phosphorylated proteins (pTyr), Hck and Nef. **C)** Fifteen initial hits from the chemical library screen were retested over a range of concentrations for rescue of growth arrest in comparison to A-419259 (5 μM). The plot shows a ranking of the results as a percentage of the growth reversion observed with A-419259. Optimal concentrations varied between compounds, which most likely reflects an effect on the Nef:Hck target vs. cytotoxicity at higher concentrations for some compounds. Data shown were obtained at 30 μM with the exception of compounds 3 and 10 (10 μM), 4 and 6 (3 μM), and 9 (1 μM).

Because Nef-induced activation of Hck-YEEI causes growth arrest, we predicted that inhibitors of this complex should restore growth, thus providing the basis for an inhibitor screen. We tested this idea with the pyrrolopyrimidine compound A-419259, a potent inhibitor of Hck and other SFKs [37-39]. Liquid cultures of yeast co-expressing Hck-YEEI and Nef were grown in the presence or absence of A-419259, and growth was monitored as the change in optical density at 600 nm. As shown in Figure 4A, A-419259 rescued growth suppression by the Nef:Hck-YEEI complex at both 1 and 5 μM in comparison to untreated cultures. At 5 μM, A-419259 treatment was nearly as effective as mutation of the Nef PxxP motif essential for SH3 binding in terms of reversing growth arrest. This effect of A-419259 correlated with a decrease in tyrosine phosphorylation of yeast proteins to control levels in the inhibitor-treated cultures (Figure 4B). The ability of A-419259 to reverse the growth arrest induced by the Nef:Hck-YEEI complex suggested that the yeast-based system may be useful for the identification of selective inhibitors of Nef:SFK signaling.

Yeast cultures expressing the Nef:Hck-YEEI complex were then used to screen a chemical library of 2496 discrete heterocyclic compounds. In the first pass, each compound was tested in duplicate at 10 μM for its ability to increase the growth of Nef:Hck-YEEI cultures relative to controls incubated with the carrier solvent alone. From this primary screen, 170 compounds were observed to restore growth of Nef:Hck-YEEI cultures by at least 10% over untreated controls. These compounds were then re-screened at 10 μM in comparison to 5 μM A-419259, the control SFK inhibitor described above. Of these, fifteen compounds were observed to rescue growth to at least 25% of the values observed with A-419259-treated positive controls. Each of these compounds was then re-purchased and tested a third time over a range of concentrations to verify growth recovery of Nef:Hck-YEEI cultures compared with A-419259. Figure 4C shows the resulting rank order of these compounds relative to the A-419259 control response. Though the activities of these compounds were lower than those observed with the original library, the rank order of their activities remained the same.

### Hit compounds from the Nef:Hck-YEEI yeast screen block Nef-dependent HIV replication

**Figure 5:**
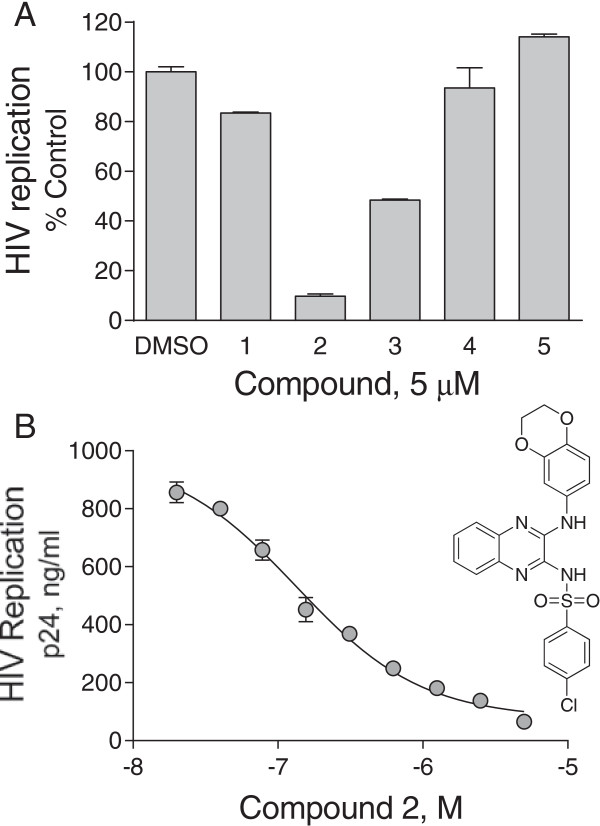
**Hit compounds from the yeast-based Nef:Hck screen block HIV replication. A)** U87MG/CD4/CXCR4 cells were infected with HIV strain NL4-3 in the presence of the top five compounds selected from the Nef:Hck-YEEI yeast screen shown in Figure 4C. Cells treated with the carrier solvent alone (DMSO) served as control. Release of viral p24 was determined in duplicate by ELISA four days post-infection, and the values shown reflect the mean percent of control ± S.D. **B)** Dose response curve for the anti-HIV activity of compound 2 from part A. Non-linear curve fitting was used to estimate an IC_50_ value of 130 nM for this compound, which is a dihydrobenzo-1,4-dioxin-substituted analog of N-(3-aminoquinoxalin-2-yl)-4-chlorobenzenesulfonamide (DQBS; structure shown).

We next evaluated hit compounds from the yeast screen for activity in a Nef-dependent HIV replication assay. For these experiments, we used U87MG astroglioma cells engineered to express the HIV-1 co-receptors CD4 and CXCR4. Replication of HIV-1 NL4-3 is dependent upon an intact viral *nef* gene in these cells, making them an ideal system to evaluate leads from our Nef-directed screen [40]. U87MG cells were infected with HIV-1 in the presence of the top five compounds identified in the yeast screen (Figure 4C) and HIV replication was monitored as p24 Gag levels by ELISA. As shown in Figure 5A, compounds 2 and 3 significantly suppressed HIV replication at a concentration of 5 μM. Neither of these compounds was cytotoxic to U87MG cells up to 50 μM, as judged by Alamar Blue (resazurin) cell viability assay, indicating that the inhibition of HIV replication is not due to non-specific effects on cell growth (data not shown). Subsequent concentration-response studies revealed that compound 2, a dihydrobenzo-1,4-dioxin-substituted analog of N-(3-aminoquinoxalin-2-yl)-4-chlorobenzenesulfonamide (DQBS; see Figure 5B for structure), potently blocked HIV replication with an IC_50_ value of 130 nM in this system (Figure 5B). Because of the remarkable potency of this compound against Nef-dependent HIV-1 replication, we explored its mechanism of action in more detail as described below.

**Figure 6:**
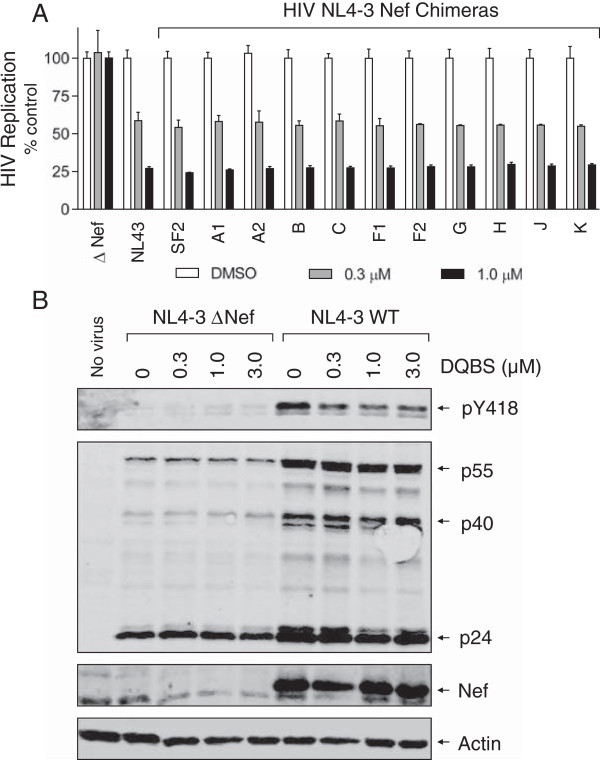
**Inhibition of HIV-1 Nef chimera replication and endogenous SFK activation by DQBS. A)** CEM-T4 cells (1 × 10^4^ per well of a 96-well plate) were infected with wild-type HIV-1 NL4-3, a Nef-defective mutant (ΔNef), or the indicated HIV-1 Nef chimeras in a final culture volume of 200 μl. Input virus for HIV-1 ΔNef was increased by ten-fold relative to wild-type to compensate for the reduced infectivity and replication of Nef-defective virus in CEM-T4 cells [41]. DQBS was added to the cultures to final concentrations of 0.3 and 1.0 μM, and viral replication was determined by p24 ELISA 10 days later. Data are expressed as the mean percent of HIV-1 replication observed in control cultures incubated with the carrier solvent (0.1% DMSO) ± S.D. from duplicate experiments performed in triplicate. **B)** CEM-T4 cells were infected with wild-type or Nef-defective (ΔNef) HIV-1 NL4-3 in the presence of the indicated concentrations of DQBS or the carrier solvent (DMSO). SFK proteins were immunoprecipitated from infected cell lysates and immunoblotted with an antibody specific for the phosphorylated activation loop tyrosine (pY418) common to all Src-family members. Controls blots were performed on the cell extracts for HIV-1 Gag proteins (p55, p40, p24), Nef, as well as actin. Blots from uninfected, untreated cells were also included as a negative control (No virus).

We next investigated whether DQBS is active against Nef proteins representative of the majority of HIV-1 M-group clades. For these studies, we first resynthesized DQBS as described under Materials and Methods, and confirmed its structure by mass spectrometry and NMR. We then tested the activity of newly synthesized DQBS in replication assays with a set of HIV-1 NL4-3 chimeras. In these HIV-1 recombinants, the NL4-3 Nef sequence is substituted with Nef sequences from HIV-1 subtypes A1, A2, B, C, F1, F2, G, H, J, K, as well as the B-clade laboratory strain, SF2 [41]. This experiment was performed in the T-cell line CEM-T4, in which HIV-1 replication is also Nef-dependent [41]. Figure 6 shows that DQBS inhibited the replication of wild-type HIV-1 NL4-3 as well as all eleven Nef chimeras with an IC_50_ value of about 300 nM. In contrast, DQBS did not affect replication of Nef-defective HIV-1 (ΔNef), supporting a Nef-dependent mechanism of action.

Because DQBS was identified as an inhibitor of Nef-dependent SFK activation, we next explored whether it affected Nef-dependent activation of endogenous SFK activity in the context of HIV-1 infection. For these experiments, CEM-T4 cells were infected with wild-type or Nef-defective HIV-1 over a range of DQBS concentrations. Endogenous SFK proteins were then immunoprecipitated from the infected cell lysates, and immunoblotted with a phosphospecific antibody against the activation loop phosphotyrosine (pY418). As shown in Figure 6B, HIV-1 infection resulted in Nef-dependent SFK activation loop tyrosine phosphorylation, and this effect was inhibited by about 50% in the presence of DQBS. This result shows that DQBS interferes with this Nef-dependent signaling function as part of its mechanism of action.

### DQBS inhibits Nef-mediated MHC-I downregulation

**Figure 7:**
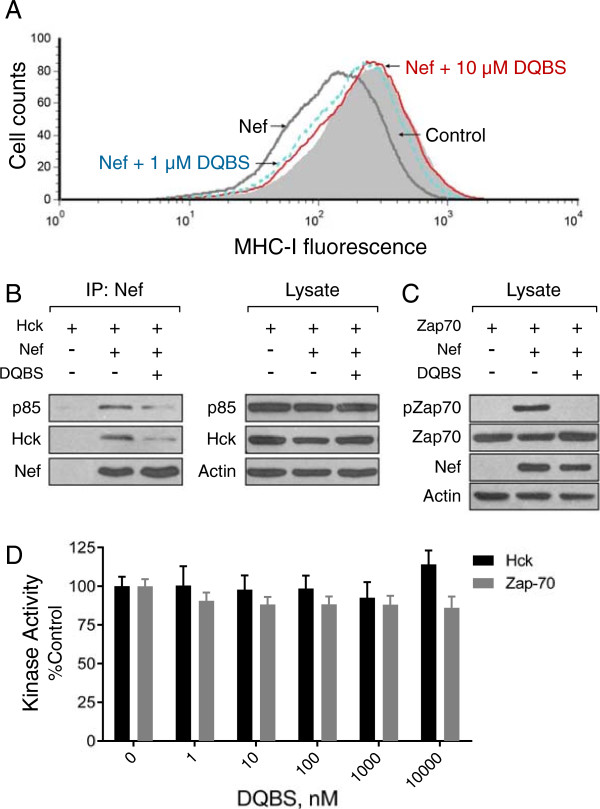
**DQBS inhibits Nef-mediated downregulation of MHC-I by preventing assembly of the SFK-ZAP-70-PI3K complex. A)** MHC-I downregulation. H9 cells were infected with a recombinant vaccinia virus carrying Nef-Flag or wild-type vaccinia as a control. Cells were then treated with DQBS at concentrations of 1 μM or 10 μM for 4 h. The cells were then fixed and processed for flow cytometry using the anti-MHC-I antibody, W6/32. **B)** Nef-SFK-PI3K co-precipitation assay. H9 cells were infected with an Hck vaccinia virus either alone or together with the Nef-Flag virus, followed by treatment with 10 μM DQBS for 4 h prior to harvest. Immunoprecipitates were prepared with the M2 anti-Flag antibody, and associated Hck and p85 were detected by immunoblotting. Control blots with the cell lysates for p85, Hck and Nef are shown on the right. **C)** Zap-70 kinase activation assay. H9 cells were infected with a Zap-70 vaccinia virus either alone or together with the Nef-Flag virus, followed by treatment with 10 μM DQBS for 4 h prior to harvest. Levels of activated Zap-70 were analyzed by immunoblotting with a phosphospecific antibody for the activation loop phosphotyrosine residue (pZap-70). Control blots for Zap-70 levels, Nef and actin are also shown. **D)** DQBS does not directly inhibit Hck or Zap-70 kinase activity in vitro. Kinase assays were performed with recombinant purified Hck-YEEI and Zap-70 in the absence or presence of the DQBS concentrations indicated using the Z’Lyte method as described elsewhere [40,45]. As inhibitor controls in the kinase assay, we observed potent inhibition of Hck by the pan-SFK/Abl inhibitor dasatinib [46] and of Zap-70 by the Syk/Zap-70 inhibitor, BAY 61–3606 [47] (data not shown).

Nef induces downregulation of cell-surface MHC-I, allowing HIV-infected cells to escape immune surveillance by cytotoxic T-cells [20-22,42,43]. To investigate the effect of DQBS on Nef-mediated MHC-I downregulation, the CD4^+^ T-cell line H9 was infected with a recombinant vaccinia virus carrying Nef or with wild-type vaccinia virus and then treated with increasing concentrations of DQBS. As shown in Figure 7A, Nef expression resulted in downregulation of cell-surface MHC-I expression by flow cytometry, consistent with previous results in this system [20,44]. Remarkably, this effect was inhibited by the presence of 1 μM DQBS and completely blocked at a concentration of 10 μM.

We next explored the mechanism of the DQBS-dependent block in Nef-induced downregulation of MHC-I. An essential first step in this pathway involves Nef-mediated assembly of a multi-kinase complex including an SFK, Syk/Zap-70, and a class I PI3K [20,21]. To determine whether DQBS affected assembly of this complex, H9 cells were co-infected with recombinant Hck and Nef vaccinia viruses in the presence or absence of DQBS. Nef immunoprecipitates were then prepared and probed for associated Hck and the p85 regulatory subunit of PI3K. Figure 7B shows that DQBS treatment reduced the amount of both Hck and p85 associated with Nef. DQBS treatment also completely blocked Nef-dependent activation of Zap-70 (Figure 7C). Using an in vitro kinase assay, we were unable to detect direct inhibition of Zap-70 or Hck by DQBS (Figure 7D), suggesting that its effects on kinase activity are mediated through Nef. Taken together, these findings suggest that DQBS prevents Nef-dependent downregulation of MHC-I by interfering with assembly of the multi-kinase complex and preventing the activation of Zap-70 downstream. Inhibition of Zap-70 may also contribute to the anti-retroviral efficacy of this compound (see Discussion).

### Docking studies predict direct binding of DQBS to Nef

The results presented above demonstrate that DQBS inhibits Nef-dependent enhancement of HIV-1 replication across a broad range of Nef subtypes. This compound also blocks Nef-mediated downregulation of MHC-I by preventing assembly and activation of downstream kinase signaling by Nef. These findings suggest that DQBS may directly target conserved features of the Nef structure. To explore possible binding sites for DQBS on Nef, we performed docking studies using a crystal structure of Nef bound to a SFK SH3 domain [35] and AutoDock Vina [48]. In this structure, the Nef:SH3 complexes pack as dimers, with the dimerization interface formed between the αB helices of the two Nef molecules. Docking analyses based on the Nef dimer returned two energetically favorable binding sites for DQBS, while docking based on a single Nef monomer returned three possible binding sites. Predicted binding site residues within 4 Å of the DQBS ligand are summarized in Table 1.

**Table 1:** Docking of the small molecule Nef antagonist DQBS to HIV-1 Nef

**Binding site**	**Binding energy (kcal/mol)**	**Nef residues within 4 Å of DQBS**
**Nef dimer**		
1	−9.0	Nef subunit 1 (blue in Figure 8): Gln104, Asp108, Pro122, Asp123
		Nef subunit 2 (green in Figure 8): Gln104, Asp108, Gln107, Asp111, Leu112, Pro122, Gln125, Asn126, Tyr127
2	−8.3	Pro78, Met79, Thr80, Tyr81, Asp123, Trp124, Asn126, Leu137, Thr138, Phe129, Tyr202
**Nef monomer**		
1	−7.9	Gln104, Gln107, Gln125, Asn126, Tyr127, Thr128, Pro129, Arg134, Leu137, Tyr202
2	−7.9	Met79, Tyr82, Asn126, Leu137, Thr138, Phe139, His193, Tyr202, Phe203
3	−7.7	Leu91, Lys94, Gly95, Gly96, Leu97, Leu100, Arg106, Ile109, Leu110, Trp113

**Figure 8:**
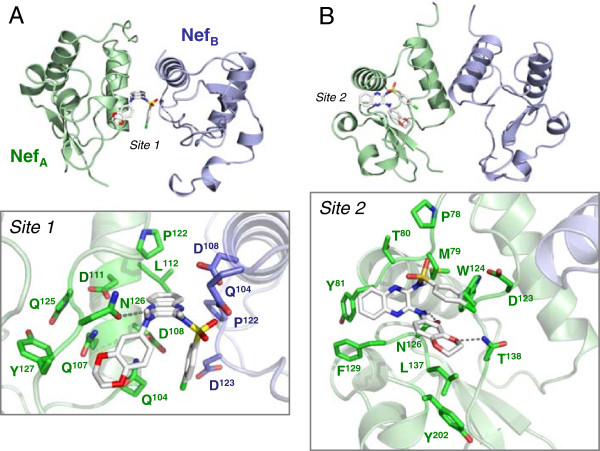
**Docking studies predict direct interaction of DQBS with HIV-1 Nef.** Molecular docking studies were performed with DQBS and the X-ray crystal structure of the Nef dimer (PDB: 1EFN). The most energetically favored sites for DQBS binding lie at the dimer interface (**A**; Site 1) and on the surface of each Nef monomer (**B**; Site 2). The location of each predicted binding site is shown on an overall view of the Nef dimer at the top with the individual Nef subunits colored in green and blue respectively. A close of up view of each binding site is shown below, highlighting the side chains of Nef residues within 4 Å of each ligand binding site. DQBS is predicted to make polar contacts with Asn126 in both binding sites, plus an additional contact with Thr138 in Site 2. For additional details of docking results, see Table 1.

Docking was performed using AutoDock Vina and an X-ray crystal structure of HIV-1 Nef as described under Materials and Methods. For the Nef dimer, two binding sites were predicted, with a preference for the dimer interface site (Site 1). Analysis using a single Nef monomer from this crystal structure returned three energetically equivalent sites. The table summarizes the binding energies and predicted binding site residues within 4 Å of the docked ligand. Molecular models for the two sites predicted from the Nef dimer are shown in Figure 8.

The most energetically favorable docking site for DQBS localizes to the Nef dimer interface, with a predicted binding energy of −9.0 kcal/mol (modeled in Figure 8A). This site involves a polar contact with Nef Asn126, a residue previously implicated in the mechanism of action of another Nef antagonist reported recently, a diphenylpyrazolo compound known as B9 [45]. In addition, this docking pose places DQBS in close contact with the side chain of Asp123, a residue critical for Nef function in MHC-I downregulation [49]. A recent crystal structure shows that Nef Asp123 interacts with the μ1 subunit of the clathrin adaptor protein AP-1, which is linked to later steps in the MHC-I endocytic pathway [20,50]. The second DQBS binding site based on the Nef dimer also involves polar contacts with Asn126 as well as Thr138, and comes in close proximity to Asp123 (Figure 8B).

Docking routines for DQBS based on an individual Nef subunit from the same crystal structure returned two sites with binding energies of −7.9 kcal/mol (Table 1). Both of these involve Asn126, which was also implicated in docking poses based on the dimer. A third putative DQBS binding site on Nef (−7.7 kcal/mol) involves Trp113, which is involved in Nef interaction with the SH3 domains of Src-family kinases (see Figure 3). In addition, Trp113 is essential for Nef binding to PACS-2, a trafficking protein critical to the assembly of the multi-kinase complex that initiates the Nef-dependent MHC-I downregulation pathway [44]. This aspect of the docking model is consistent with our observations that DQBS destabilizes the multi-kinase complex and prevents activation of Zap-70 in the context of Nef-induced MHC-I downregulation (Figure 7). Overall, the docking studies raise the possibility that DQBS may interact with multiple sites on Nef, providing a mechanistic basis for its potent activity against several Nef functions (see Discussion).

### Direct interaction of DQBS with Nef by differential scanning fluorimetry

**Figure 9:**
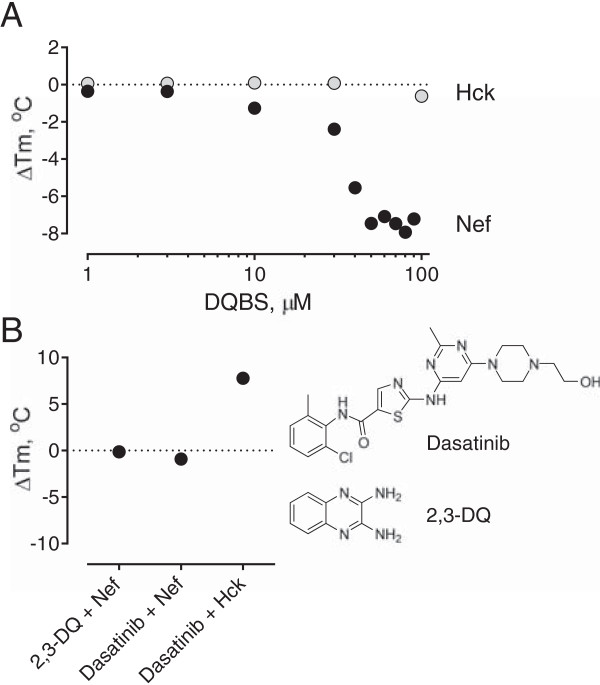
**DQBS induces thermal destabilization of Nef. A)** Differential scanning fluorimetry assays were performed using recombinant purified Nef and Hck-YEEI proteins in the presence of DQBS as described under Materials and Methods. Data are plotted as the change in the mid-point of each thermal melt profile (ΔT_m_) as a function of DQBS concentration relative to the DMSO control. **B)** ΔT_m_ values were determined for Nef and Hck-YEEI in the presence of 2,3-diaminoquinoxaline (2,3-DQ) or the kinase inhibitor dasatanib, each at a concentration of 100 μM. The chemical structures of dasatinib and the DQBS parent scaffold 2,3-DQ are shown on the right; note that 2,3-DQ is inactive in all Nef and HIV assays tested. In both **A** and **B**, each data point represents an average of 2 to 8 separate DSF experiments, each performed in triplicate.

Docking studies presented in the previous section support direct interaction of DQBS with Nef. To test this possibility experimentally, we developed a differential scanning fluorimetry assay [51,52] in which purified recombinant Nef is gradually heated in a quantitative PCR instrument in the presence of the reporter dye, SYPRO orange. As the temperature rises and Nef unfolds, the reporter dye gains access to the hydrophobic interior of the Nef protein, resulting in an increase in dye fluorescence. The resulting rise in fluorescence as a function of temperature eventually reaches a maximum, and the resulting protein ‘melt curve’ is fit by non-linear regression analysis to obtain a T_m_ value (temperature at which half-maximal thermal denaturation is observed). For full-length recombinant Nef, we observed a very consistent T_m_ value of 61.5 ± 0.6°C. This experiment was then performed over a range of DQBS concentrations. As shown in Figure 9A, addition of DQBS resulted in a concentration-dependent decrease in the T_m_ value, with a maximum reduction of about 8°C. In contrast to Nef, DQBS had no effect on the thermal stability of recombinant, near-full-length Hck, providing additional evidence that DQBS works by interacting with Nef and not with its partner kinase. Additional control experiments show that the unsubstituted 2,3-diaminoquinoxaline pharmacophore as well as a structurally unrelated compound (the kinase inhibitor dasatinib) had no effect on the thermal stability of Nef even at concentrations as high as 100 μM (Figure 9B). Dasatinib, on the other hand, caused a dramatic increase in the thermal stability of Hck (Figure 9B), which agrees with the potent inhibition of Hck by this compound (data not shown). These new data provide important evidence that DQBS interacts directly with the Nef protein, and may destabilize its quaternary structure and/or interactions with effector proteins as a possible mechanism of action.

### Comparison of the anti-HIV activities of DQBS with other Nef antagonists

**Figure 10:**
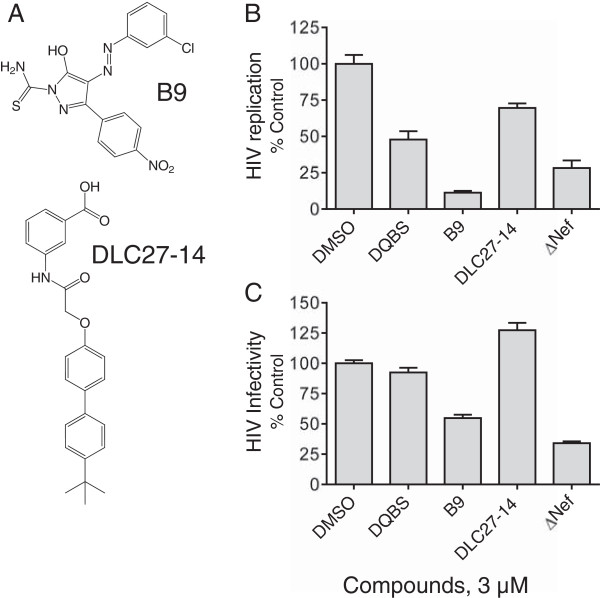
**Comparison of DQBS with other Nef inhibitors on HIV replication and infectivity. A)** Structures of the Nef inhibitors B9 and DLC27-14 (see main text for details). **B)** HIV-1 replication assays were performed in U87MG/CD4/CXCR4 cells in the absence (DMSO control) or presence of each compound as described in the legend to Figure 5. Cells infected with an equivalent p24 input of Nef-defective HIV (ΔNef) are included as a reference control. **C)** TZM-bl reporter cells, in which the HIV-1 LTR drives transcription of luciferase [56], were infected with wild-type or Nef-defective (ΔNef) HIV-1 NL4-3 in the absence (DMSO) or presence of each of the Nef inhibitors shown. Viral infectivity was assessed as luciferase activity 48 h later. This experiment was repeated in triplicate and the data are presented as mean percent infectivity relative to the DMSO control.

In addition to DQBS, a handful of other compounds have been reported to bind to Nef and impact its functions (for a review, see Smithgall and Thomas [53]). These include the diphenylpyrazolodiazene compound B9 described above, which is predicted to bind to the Nef dimerization interface [45], as well as DLC27-14, which was computationally designed to block Nef interaction with SH3 domains and may also destabilize the Nef structure [54,55]. The structures of these compounds are presented in Figure 10A. In a final series of studies, we compared their activity to that of DQBS in HIV assays that are influenced by the presence of Nef. HIV replication assays were performed in U87MG/CD4/CXCR4 cells with all compounds tested at a concentration of 3 μM. As shown in Figure 10B, both DQBS and B9 reduced viral replication to levels near or below that observed with the Nef-defective virus, consistent with data presented above for DQBS and in previous studies for B9 [45]. On the other hand, DLC27-14 was less potent, reducing viral replication by about 25% at this concentration. Each of these compounds was also tested in the TZM-bl reporter cell line [56] for effects on early events in the viral life cycle. Interestingly, only B9 inhibited viral infectivity and gene expression at this concentration, consistent with published results [45]. While cytotoxicity precluded the evaluation of DQBS at higher concentrations in this assay, these results suggest that it may act at later stages of the viral life cycle.

## Discussion

In this report we describe the discovery of a unique antagonist of the HIV-1 accessory protein, Nef, using a yeast-based screening assay. This assay exploits the growth-suppressive actions of Src-family kinases on yeast cell growth [26,31]. In our case, we engineered yeast strains to co-express Nef and the Src-family kinase Hck, one of the best-characterized Nef target proteins. Nef interacts with Hck and switches on its kinase activity by binding to its SH3 domain, resulting in growth arrest. Hit compounds were selected based on their ability to rescue growth suppression by the Nef:Hck complex. One advantage of this approach is that non-selective cytotoxic compounds cannot rescue growth and therefore do not score as false positives. Remarkably, two of the top five compounds identified in the yeast screen were subsequently found to block Nef-dependent HIV-1 replication in vitro. One of these, the 2,3-diaminoquinoxaline analog DQBS, not only blocked Nef-dependent HIV-1 replication with submicromolar potency across a wide spectrum of Nef subtypes, but was also shown to reverse MHC-I downregulation by Nef.

DQBS was isolated from a chemical library biased towards heterocyclic structures that resemble protein kinase inhibitors, raising the possibility that it may target the ATP-binding site of Nef-activated SFKs or Zap-70 rather than Nef directly. However, using an in vitro kinase assay and recombinant purified Hck and Zap-70, we were unable to detect direct inhibition of kinase activity by DQBS. A more likely mechanism of action for DQBS involves direct interaction with Nef, thereby interfering with recruitment and activation of SFKs and other Nef effector proteins. This possibility is supported by docking studies, which predicted several energetically favorable binding sites for DQBS on the Nef structure. Remarkably, several of the Nef residues predicted to interact with DQBS have been previously identified in a similar docking study of the Nef antagonist, B9, including Gln104, Gln107, and Asn 126 [45]. The observation that two independent screens yielded Nef antagonists with overlapping predicted binding sites suggests that this region may represent a hot spot for Nef inhibitor development. Direct interaction of DQBS with Nef is supported by differential scanning fluorimetry assays presented here, which showed that this compound causes thermal destabilization of the Nef protein in a concentration-dependent manner.

One exciting feature of DQBS is its potent activity against Nef-dependent downregulation of MHC-I, which is believed to allow HIV-infected cells to escape immune surveillance. The extent of MHC-I downregulation in simian immunodeficiency virus (SIV)-infected macaques correlates directly with the severity of disease progression, supporting a critical role for this immune evasive mechanism in vivo [57]. A possible mechanism by which DQBS blocks this critical Nef function is suggested by the way in which Nef initiates MHC-I downregulation in HIV-infected cells. During the first two days following infection, Nef triggers MHC-I downregulation by an endocytic program termed the signaling mode. By three days post-infection, Nef switches to a stoichiometric mode of downregulation to prevent newly synthesized MHC-I molecules from reaching the cell surface [20]. One early step in the signaling mode involves Nef-dependent assembly of a multi-kinase complex involving a Src-family member (Hck, c-Src or Lyn, depending upon the cell lineage), Zap-70 or Syk, and a class I PI3K [20,22]. Data presented in Figure 7 support a model in which DQBS interferes with the initial assembly of this kinase complex, resulting in a complete block in the activation of Zap-70. Control kinase assays show that DQBS does not impact Zap-70 or Hck activity directly, supporting a mechanism of action that is mediated through Nef. In addition, computational docking studies show that DQBS binding may influence the accessibility of Nef Trp113, which is required for interaction with SFK SH3 domains as well as the PACS-2 trafficking protein that triggers SFK/Zap-70/PI3K complex assembly [22,44].

Interestingly, Zap-70 has also been implicated in HIV-1 replication and viral spread [58,59], suggesting that DQBS may interfere with HIV-1 replication by blocking Nef-dependent Zap-70 activation in CEM-T4 cells and other T-cell hosts. Future work will address whether DQBS can similarly inhibit HIV-1 replication in macrophages, which express the Zap-70 homolog, Syk. The finding that Syk interchanges with Zap-70 in the Nef-assembled multi-kinase complex in cells of the monocytic lineage supports this possibility [22]. The potent inhibition of Nef-dependent HIV-1 replication in U87MG/CD4/CXCR4 cells reported here may also involve Syk, which exhibits a more widespread expression pattern than Zap70 [60]. Taken together, these findings support an inhibitory mechanism in which DQBS binds directly to Nef and interferes with its activation of a common intermediate (Zap-70 or Syk) in both the MHC-I downregulation pathway and in HIV-1 replication.

Another Nef-binding compound, the *Streptomyces* natural product derivative known as ′2c’, has also been reported to affect Nef-dependent MHC-I downregulation [20,61] and viral infectivity. NMR studies showed that 2c interacts primarily with Nef through a cleft formed by the central β-sheet and the C-terminal α-helices. While the 2c binding site is distinct from those for DQBS on Nef presented here, neither binding site overlaps with Nef structural features involved in SH3 binding and SFK recruitment. These observations suggest an allosteric mechanism of action for both compounds. Compared to DQBS, however, the potency of 2c is lower in terms of both MHC-I downregulation and antiviral activity. This difference may relate to a weaker binding affinity of 2c for Nef as well as the possibility that DQBS may occupy multiple sites on the Nef structure that are important for MHC-I downregulation as well as viral growth.

## Conclusions

Antiretroviral agents currently used for the treatment of AIDS target the viral reverse transcriptase, integrase and protease or block virus-host cell fusion [62]. Data presented here with the compound DQBS support the idea that the HIV-1 accessory protein Nef represents an alternative target for antiretroviral drug action. This compound not only inhibits enhancement of HIV replication by Nef, but also reverses Nef-mediated downregulation of MHC-I, raising the exciting possibility that it may enhance recognition of HIV-infected cells by cytotoxic T-cells. The growing number of HIV strains resistant to conventional antiretroviral therapy [63,64] combined with the lack of an HIV vaccine underscore the need for new anti-HIV drugs. Work presented here shows that compounds targeting HIV-1 Nef may provide a new avenue for anti-HIV therapy, and demonstrates the potential of a yeast-based, phenotypic screen based on the complex of an HIV-1 accessory protein with a host cell kinase as a route to their discovery.

## Methods

### Yeast expression vectors

Coding sequences for human Csk and Hck as well as HIV-1 Nef (SF2 strain) were modified by PCR to introduce a yeast translation initiation sequence (AATA) immediately 5′ to the ATG start codon. The coding sequence for Hck was subcloned downstream of the Gal10 promoter in the pYC2/CT vector (Invitrogen), which carries the CEN6/ARSH4 sequence for low-copy replication. The Csk and Nef coding sequences were subcloned downstream of the Gal1 and Gal10 promoters, respectively, in the yeast expression vector pESC-Trp (Stratagene). The coding sequence of the wild-type Hck tail (YQQQP) was modified by PCR to encode the high-affinity SH2-binding sequence, YEEIP, as described elsewhere [32,65]. The Nef-PA mutant, in which prolines 72 and 75 are replaced with alanines, has also been described elsewhere [17].

### Yeast growth suppression assay

*S. cerevisiae* strain YPH 499 (Stratagene) was co-transformed with pESC-Ura (or pYC2/CT) and pESC-Trp plasmids containing the genes of interest via electroporation (BioRad Gene Pulser II). Yeast were selected for three days at 30°C on standard synthetic drop-out plates lacking uracil and tryptophan (SD/-U-T) with glucose as the sole carbon source to repress protein expression. Positive transformants were grown in liquid SD/-U-T medium plus glucose, normalized to OD_600nm_ = 0.2 in water, and then spotted in four-fold dilutions onto SD/-U-T agar plates containing galactose as the sole carbon source to induce protein expression. Duplicate plates containing glucose were also prepared to control for yeast loading (data not shown). Plates were incubated for three days at 30°C and imaged on a flatbed scanner. Yeast patches appear as dark spots against the translucent agar background. All growth suppression assays were repeated at least three times starting with randomly selected independent transformed clones and produced comparable results; representative examples are shown. For the liquid growth assay, yeast strain W303a (gift of Dr. Frank Boschelli, Wyeth Pharmaceuticals) was co-transformed with the required plasmids, seeded at an initial density of OD_600nm_ = 0.05 units in SD/-U-T medium, and incubated for 21 h at 30°C. The control inhibitor A-419259 was added with DMSO as carrier solvent to a final concentration of 0.1%.

### Immunoblotting from yeast cultures

Aliquots of the yeast cultures used for the spot assay were grown in SD/-U-T medium plus galactose for 18 h. Cells were pelleted, treated with 0.1 N NaOH for 5 min at room temperature [66], and normalized with SDS-PAGE sample buffer to 0.02 OD_600nm_ units per μl. Aliquots of each lysate (0.2 OD_600nm_ units) were separated via SDS-PAGE, transferred to PVDF membranes, and probed for protein phosphotyrosine content with a combination of the anti-phosphotyrosine antibodies PY99 (Santa Cruz Biotechnology) and PY20 (Transduction Laboratories). Immunoblots were also performed with antibodies to Csk (C-20; Santa Cruz), Hck (N-30; Santa Cruz), actin (MAB1501; Chemicon International) and Nef (monoclonal Hyb 6.2; NIH AIDS Research and Reference Reagent Program).

### Yeast inhibitor screen

Yeast strain W303a was co-transformed with Hck-YEEI and Nef expression plasmids and grown to an OD_600nm_ of 0.05. Cells (100 μl) were plated in duplicate wells of a 96-well plate in the presence of each compound from the ChemDiv kinase-biased inhibitor library (ChemDiv, Inc., San Diego, CA). All compounds were initially screened at 10 μM with 0.5% DMSO as carrier solvent. Control wells contained 0.5% DMSO to define the extent of growth arrest as well as cells transformed with Hck-YEEI plus the Nef-2PA mutant to define maximum outgrowth. Each plate also contained wells with 5 μM A-419259 as a positive control for drug-mediated growth reversion. Cultures were incubated at 30°C, and the OD_600nm_ was measured at 0 and 22 h. Those compounds which induced a 10% or greater increase in yeast growth relative to the DMSO control were further assayed in triplicate and compared against A-419259-mediated growth reversion. Compounds from this secondary screen which recovered yeast growth to at least 25% of that observed with A-419259 were obtained in powder form from the provider of the original library (ChemDiv) and assayed a third time in triplicate at 1, 3, 10, and 30 μM in comparison with 5 μM A-419259.

### HIV assays

HIV-1 replication assays were conducted using the HIV-1 strain NL4-3. Viral stocks were prepared by transfection of 293 T cells (ATCC) with proviral genomes for the wild-type, Nef-defective (ΔNef), or Nef chimeras (all based on NL4-3 backbone) and amplified in the T-cell line, MT2 (NIH AIDS Research and Reference Reagent Program) as previously described [41,45]. Viral replication was assessed in the U87MG astroglioma cell line engineered to express the HIV-1 co-receptors CD4 and CXCR4 or in the T-lymphoblast cell line, CEM-T4 [41,45]. Both the U87MG and CEM-T4 cell lines support HIV-1 replication in a Nef-dependent manner, and were obtained from the NIH AIDS Research and Reference Reagent Program. Compounds were solubilized in DMSO, and added to the cell culture medium 1 h prior to infection with HIV. Viral replication was monitored for either 4 days (U87MG) or 9 days (CEM-T4) by measuring p24 Gag protein levels in the culture supernatant using standard ELISA-based techniques. HIV-1 infectivity was measured using the reporter cell line TZM-bl, in which the HIV LTR drives transcription of luciferase [56]. Details of the assay conditions are described elsewhere [45].

### Activation of endogenous SFKs by HIV-1 Nef

CEM-T4 cells (1 × 10^5^) were infected with 50 pg p24 equivalents/ml of wild-type HIV-1 NL4-3 or the Nef-defective mutant in a final culture volume of 10 ml. DQBS or the DMSO carrier solvent alone were added followed by incubation for eight days. The infected cells were then lysed in RIPA buffer and endogenous Src-family kinases were immunoprecipitated with a pan-specific antibody and protein-G Sepharose beads as described elsewhere [41]. Kinase activation was assessed by immunoblotting each immunoprecipitate with a phosphospecific antibody against the activation loop phosphotyrosine residue (pY418) common to all Src family members. Control blots were performed on cell lysates for HIV-1 Gag proteins (p55, p40, and p24), Nef, as well as actin as a loading control.

### MHC-I downregulation assays

H9 T cells were infected with wild-type vaccinia virus or with a vaccinia recombinant expressing Nef-Flag (moi = 10) for 8 h as described previously [22,42]. Cells were incubated in the presence of DQBS or carrier solvent alone (DMSO) for 4 h prior to harvest. The cells were then fixed in 2% paraformaldehyde, washed and resuspended in FACS buffer (PBS, pH 7.2, containing 0.5% FBS) and incubated with mAb W6/32 (anti-MHC-I, 1:4,000) followed by PE-conjugated donkey anti-mouse IgG (1:1,000; Jackson IR, West Grove, PA). Cells were analyzed by listmode acquisition on a FACSCalibur (BD) flow cytometer using CellQuest acquisition/analysis software (BD) and data analyzed using CellQuest or FCS express (De Novo Software, Los Angeles, CA).

### Co-immunoprecipitation of Nef:kinase complexes

To measure the effect of DQBS on the interaction between Nef, Hck and class I PI3K, H9 T cells were co-infected with wild-type vaccinia virus (moi = 10) or a combination of the Nef-Flag (moi = 10) and Hck viruses (moi = 12 total). Cells were treated with 10 μM DQBS at 4 h post-infection and harvested 4 h later by lysis in PBS containing 1% NP40 supplemented with protease and phosphatase inhibitors. Nef-Flag was immunoprecipitated with mAb M2-agarose beads (Sigma) and co-immunoprecipitating Hck (N-30, Santa Cruz) and p85 (Millipore) were detected by immunoblot analysis. Nef-Flag recovery was confirmed by immunoblotting with anti-Nef antibodies (AIDS Reagent and Reference Program). Control blots of cell lysates were performed with actin antibodies (mAb 1501, Millipore). To measure the effect of DQBS on the Nef-dependent activation of Zap-70, H9 cells were co-infected with wild-type vaccinia virus (moi = 6) or the Nef-Flag (moi = 6) and Zap-70 viruses (moi = 10 total). Infected cells were then treated with 10 μM DQBS for 4 h prior to harvest and lysed as described above. The presence of active ZAP-70 was assessed by immunoblotting with a phosphospecific antibody against the activation loop phosphotyrosine site (pY319-ZAP-70; clone 2 F3.2, Millipore). Zap-70 (Cell Signaling) and Nef levels were measured by immunoblotting of the clarified cell lysates.

### Molecular docking

The structure of DQBS was docked to the crystal structure of HIV-1 Nef [35] (PDB: 1EFN; without the SH3 domain) using AutoDock Vina [48]. Independent docking routines were performed using the Nef dimer and a single Nef monomer. The three-dimensional structures of the compound and the Nef proteins were first converted from pdb into pdbqt format with MGL Tools [67]. The Nef structures were kept rigid during the docking routine, while rotatable bonds in DQBS imparted ligand flexibility. A grid box was centered on and covered each Nef structure. Nef residues predicted to participate in Nef:DQBS complex formation from the docking results with the lowest binding energies are presented in Table 1.

### Synthesis of DQBS

The synthesis of all compounds was performed under a nitrogen atmosphere. Commercially available precursors, solvents and reagents (Aldrich) were used without additional purification. NMR spectra were recorded on a Bruker 600 MHz spectrometer; chemical shifts are given in ppm and are referenced to residual solvent peaks.

#### 4-Chloro-N-(3-chloro-quinoxalin-2-yl)-benzenesulfonamide (QBS)

4-Chlorobenzenesulfonamide (1.92 g, 10 mmol) was dissolved in anhydrous DMF (50 ml). Potassium carbonate (1.38 g, 10 mmol) was added in one portion, and the reaction mixture was stirred for 10 min. 2,3-Dichloroquinoxaline (1.99 g, 10 mmol) was added, and the reaction mixture was refluxed under N_2_ for 2.5 h with reaction progress monitored by TLC (hexanes/ethyl acetate 3:1 as mobile phase). The reaction mixture was cooled and added slowly to an aqueous solution of acetic acid (1%, 500 ml) with vigorous stirring. The product precipitated as grey crystals, which were filtered and dried overnight in a desiccator (Drierite). Yield 2.32 g, 66%. R_f_ = 0.7 (hexanes/ethyl acetate 1:1).

#### 4-Chloro-N-[3-(2,3-dihydrobenzo [1,4] dioxin-6-ylamino)-quinoxalin-2-yl]-benzenesulfonamide (DQBS)

Compound QBS (354 mg, 1 mmol; above) was dissolved in xylenes (20 ml). 6-Amino-1,4-benzodioxane (2 mmol, 246 μl) was added and the reaction mixture was refluxed under N_2_ for 5 h. The solvent was evaporated under vacuum, and DQBS was isolated and purified by column chromatography (hexanes/ethyl acetate 9:1 as solvent phase). The final product formed yellow crystals with a melting point of 257-258°C. Yield, 61%. R_f_ = 0.3 (hexanes/ethyl acetate 3:1). ^1^H NMR (CDCl_3_, 600 MHz): δ 4.31 (m, 2H), 6.88 (d, *J* = 9.0 Hz, 1H), 7.15 (dd, *J* = 9.0 Hz, 2.4 Hz, 1H), 7.29 (dd, *J* = 1.2 Hz, 1H), 7.36 (td, *J* = 7.8 Hz, 1.2 Hz, 1H), 7.42 (td, *J* = 7.8 Hz, 1.2 Hz, 1H), 7.53 (d, *J* = 9 Hz, 2H), 7.70 (m, 2H), 7.98 (d, *J* = 8.4 Hz, 2H), 8.19 (br.s, 1H), 11.88 (br.s, 1H). ^13^C NMR (CDCl_3_, 150 MHz): δ 64.34, 64.53, 109.36, 113.54, 116.18, 117.28, 124.16, 125.87, 126.60, 126.81, 127.89, 129.38, 131.99, 134.18, 139.41, 140.14, 140.28, 141.24, 143.43, 144.08. HRMS [C_22_H_18_ClN_4_O_4_S]^+^: calculated, 469.0732; observed 469.0704.

### Differential Scanning Fluorimetry (DSF)

A real-time StepOnePlus qPCR instrument (Applied Biosystems) and software (version 2.3) were used to perform DSF measurements. Recombinant full-length Nef (SF2 allele) and human Hck-YEEI were expressed and purified as described previously [40,41]. DSF assays (20 μl) were run in triplicate wells in MicroAmp Fast 96-well qPCR plates sealed with optical adhesive covers (Applied Biosystems). Baseline DSF profiles were obtained with recombinant Nef and Hck-YEEI proteins (1 μM) in bicine buffer (10 mM bicine, 150 mM NaCl, pH 8.0) and SYPRO Orange (Sigma) diluted to a 5X working concentration as described [51,52]. The test compounds DQBS, 2,3-diaminoquinoxaline (ChemDiv) and dasatinib (LC Laboratories) were solubilized in DMSO and diluted into the DSF assays, followed by incubation for 15 min with each protein at 4°C prior to the addition of SYPRO Orange. Parallel reactions were run in the absence of the proteins to correct for background fluorescence. The final DMSO concentration in all reactions was 1.1%. For DSF measurements, the qPCR instrument was set to use the ROX emission filter (≅ 610 nm) without a quencher or passive reference as recommended by the manufacturer. DSF mixtures were allowed to equilibrate to 25°C for 2 min, followed by an increase to 99°C at a 1% temperature ramp rate (1.6°C/min) with continuous data collection. Data were corrected for background (no protein controls) and mean fluorescence intensities were plotted as a function of temperature. The resulting melt curves were fit to the Boltzmann sigmoid function using GraphPad Prism 6, and melt temperature (T_m_) values were derived from the midpoint of the melt transition as described previously [51,52]. ΔT_m_ values were calculated as the difference between the T_m_ values obtained in the presence and absence of each test compound.

## Competing interests

The authors declare that they have no competing interests.

## Authors’ contributions

RPT performed all yeast experiments and drafted the manuscript. PN performed the virus replication assays with the HIV Nef chimeras. LES performed HIV and in vitro kinase assays. JJA developed the differential scanning fluorimetry assay for Nef and determined the T_m_ values for control and test compounds. KA investigated the effect of DQBS on the multi-kinase complex related to MHC-I downregulation. LT performed the flow cytometry studies for MHC-I downregulation. TK performed initial HIV assays on the hit compounds from the yeast screen. NY performed the computational docking studies. VK synthesized and characterized DQBS. BD participated in the design of the synthetic route to DQBS. GT participated in the design of the MHC-I downregulation studies. TES conceived the study, participated in the design and coordination of the experiments, co-wrote and edited the manuscript. All authors have read and approved the final manuscript.
